# Automated *in vitro* evolution of a translation-coupled RNA replication system in a droplet flow reactor

**DOI:** 10.1038/s41598-018-30374-0

**Published:** 2018-08-08

**Authors:** Tomoaki Yoshiyama, Tetsuo Ichii, Tetsuya Yomo, Norikazu Ichihashi

**Affiliations:** 1 Graduate School of Information Science and Technology, Osaka University, Osaka, Japan; 2 Exploratory Research for Advanced Technology, Japan Science and Technology Agency, Tokyo, Japan; 3 Institute of Biology and Information Science, East China Normal University, 3663 Zhongshan North Rd., Shanghai, 200062 P.R. China; 4 Graduate School of Frontier Biosciences, Osaka University, Osaka, Japan

## Abstract

Automation is a useful strategy to make laborious evolutionary experiments faster and easier. To date, several types of continuous flow reactors have been developed for the automated evolutionary experiments of viruses and bacteria. However, the development of a flow reactor applicable to compartmentalized *in vitro* self-replication systems is still a challenge. In this study, we demonstrate automated *in vitro* evolution of a translation-coupled RNA system in a droplet flow reactor for the first time. This reactor contains approximately 10^10^ micro-scale droplets (average diameter is approximately 0.8 μm), which continuously fuse and divide among each other at a controllable rate. In the droplets, an RNA (artificial genomic RNA) replicate through the translation of self-encoded RNA replicase with spontaneously appearing parasitic RNA. We performed two automated replication experiments for more than 400 hours with different mixing intensities. We found that several mutations displayed increased frequencies in the genomic RNA populations and the dominant RNA mutants acquired the ability to replicate faster or acquired resistance to the parasitic RNA, demonstrating that Darwinian evolution occurred during the long-term replication. The droplet flow reactor we developed can be a useful tool to perform *in vitro* evolutionary experiments of translation-coupled systems.

## Introduction

One useful method to understand complicated biological phenomena is to reconstitute these systems from a set of molecules *in vitro*. *In vitro* reconstitution allows us to understand the design principles for the target biological phenomena and may also give rise to new technologies^1–10^. To date, various basic biological functions have been reconstituted, including genome replication, gene expression, membrane-associated functions, and others as recently reviewed^11–13^. Recently, higher-order biological phenomena, such as cell division machinery^14^, predator-prey oscillation^15^, DNA replication-cell division coupling^16^, and translation-coupled replication and evolution^17^ have being reconstituted from the combination of basic biological functions.

Evolution is another complex biological phenomenon that could be understood by *in vitro* reconstitution. Natural organisms evolve through spontaneous mutations and natural selection during a long term process of self-reproduction. A similar evolutionary process was simulated *in vitro* by Spiegelman’s group in 1967^18^. Indeed, they repeated the replication of a genomic RNA of the bacteriophage Qbeta in the presence of RNA replicase through a long serial transfer experiment and observed the evolution of smaller RNAs. Later, other self-replication systems of RNA or DNA were constructed and evolution was observed in some cases^19–21^.

Recently, we constructed a translation-coupled RNA replication system by combining a cell-free translation system with Spiegelman’s RNA replication system^22^. In this system, an artificial genomic RNA replicates via the translation of a self-encoded RNA replicase. In contrast to the previous system that did not include translation, this system requires cell-like compartments for evolution to ensure the linkage between genotype (RNA) with phenotype (replicase)^17^ and to repress spontaneously appearing parasitic RNAs^23^. In the previous study, we used water-in-oil droplets for cell-like compartments, which had been used to compartmentalize various biochemical reactions^24,25^.

One of the largest hurdles in performing evolution experiments is the requirement of laborious experimental processes for long durations. To overcome this obstacle, automated systems, such as continuous flow stirred tank reactors (CSTRs) or chemostats, have been developed to examine the experimental evolution of bacteriophages^26–28^, bacteria^29–31^, fungi^32^, and artificial self-replicating DNAs^33^. However, an automated system applicable to a compartmentalized *in vitro* system has yet to be established.

Recently, we developed a continuous droplet flow reactor in which oil and water phases are continuously supplied into a chamber^34,35^. In this system, the contents of the droplets were continuously diluted with the supplied water phases through fusion and division of the droplets induced by mixing. This type of the exchange of contents of water-in-oil droplets has been utilized to induce internal biochemical reactions in previous studies^36–38^. In our previous report, we succeeded in performing translation-coupled RNA replication in this droplet flow reactor for several hours^35^. However, the duration of the RNA replication was not sufficient to observe evolution.

In this study, we attempted to establish automated *in vitro* RNA evolution in the droplet flow reactor. We carefully adjusted several parameters in the droplet flow reactor and successfully performed two long-term replication experiments for more than 400 hours each. Mutations accumulated in the RNAs during replication and the RNAs in the final populations acquired the ability to replicate faster or developed a tolerance to the parasitic RNA.

## Results

### Translation-coupled RNA replication system

**Figure 1:**
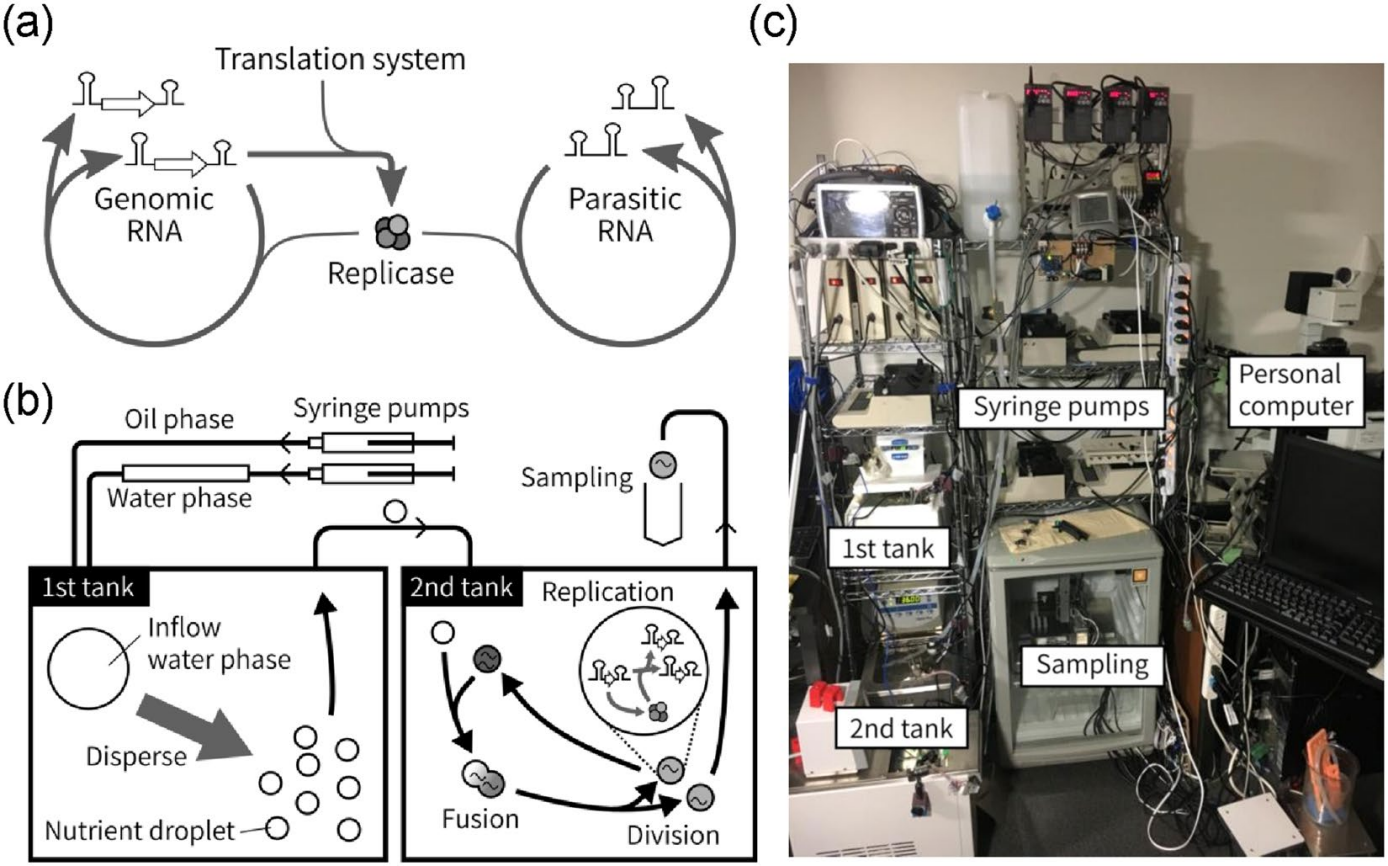
Reaction scheme of the translation-coupled RNA replication in a continuous droplet flow reactor. (**a**) Reaction scheme of the translation-coupled RNA replication. The host genomic RNA replicates using the replicase translated from itself. The parasitic RNA, which does not encode replicase gene, replicates relying on the replicase translated from the host RNA. (**b**) A continuous droplet flow reactor. The oil phase and the water phase containing the translation system were supplied to the first tank, in which water-in-oil droplets were produced by mixing. The droplets were supplied to the second tank, where RNA replication and fusion/division among droplets occur. The second tank contained approximately 10^10^ droplets of 0.8 μm average diameter. The droplets in the second tank were diluted at a certain rate (dilution rate) with the droplets supplied from the first tank to maintain the total number of droplets. The mixing intensity in the tanks was controlled by the mixing period (duty ratio) of magnetic stirrer bars inside. (**c**) A picture of the droplet flow reactor.

The translation-coupled RNA replication system used in this study consists of an artificial genomic RNA and a reconstituted translation system of *E. coli*. The genomic RNA encodes the catalytic subunit of an RNA replicase (Qbeta replicase), which is translated and forms an active replicase with EF-Tu and EF-Ts in the translation system. The replicase catalyzes the replication of the genomic RNA. During replication, parasitic RNAs, which have lost the replicase gene, appeared spontaneously, possibly through recombination^39,40^. These RNAs replicate using the replicase translated from the genomic RNA (Fig. 1a). Therefore, the genomic RNA works as a “host” for the parasitic RNA. This system was encapsulated in water-in-oil droplets of a few micro-meter diameters. In our previous studies, we manually performed a long-term replication with a temporal supply of new droplets containing the translation system^17,23^, which took significant effort and time to observe the evolution of the genomic RNAs.

### Droplet flow reactor

In this study, we modified the droplet flow reactor we have constructed^34,35^ to perform RNA replication automatically for multiple generations,. This system consists of two tanks and the mixing rates are controlled by changing the rotation frequency of the magnetic stirrer bars in both tanks (Fig. 1b,c). Oil and aqueous phases were supplied to the first tank with syringe pumps. The oil phase contains mineral oil and surfactants (Span 80 and Tween 80) and the aqueous phase contains the *E. coli* translation system, including NTPs. In the first tank, nutrient droplets that contain the translation system were prepared. The nutrient droplets were supplied to the second tank, where fusion and division among the droplets were induced by controlling the mixing rates with the magnetic stirrer bar. Through this process, the contents of most of the droplets were well mixed as shown by the dispersion of the fluorescent dye in the droplets (Fig. S1). In the second tank, some droplets contained the genomic and/or parasitic RNAs, and RNA replications occurred at 36 °C. Through this fusion-division process, the genomic and parasitic RNAs propagate into other droplets and continue to replicate. The droplets were removed from the second tank at the same rate as the supply rate of the nutrient droplets (i.e., dilution rate). During replication, mutations are spontaneously introduced by replication error into the RNAs^17^, and if a more replicable RNA mutant appears, it will dominate the population according to Darwinian principles.

### Long-term replication experiments

In previous report, we have succeeded in replication for only few hours in the droplet flow reactor. We believe that this was caused by a high mixing intensity in the second tank, which was uncontrollable in the previous system. In this study, we modified the control program to make the mixing intensity controllable. After several trials with different mixing intensities and dilution rates, we have successfully performed long-term replication experiments for 412–421 hours at two different (low or high) mixing rates in the second tank. The key factors that are important for sustainable replication was described in the Methods section. The low and high mixing rates were conducted by changing the working period of the stirrer in the second tank (duty ratio) at 5% or 20%, respectively. The size of droplets was not significantly changed with these duty ratios (Fig. S2). During the experiment, we measured the genomic and parasitic RNA concentrations every hour for 24 hours. Genomic RNA was measured by quantitative PCR after reverse transcription. This method is highly sensitive and we were able to measure with sensitivity greater than 10^−5^ nM. The parasitic RNA was not measurable by this method due to the presence of secondary structures, and thus, quantified using the band intensities after PAGE followed by RNA staining with SYBR green II. The detection limit of this method is much higher (approximately 3 nM).

**Figure 2:**
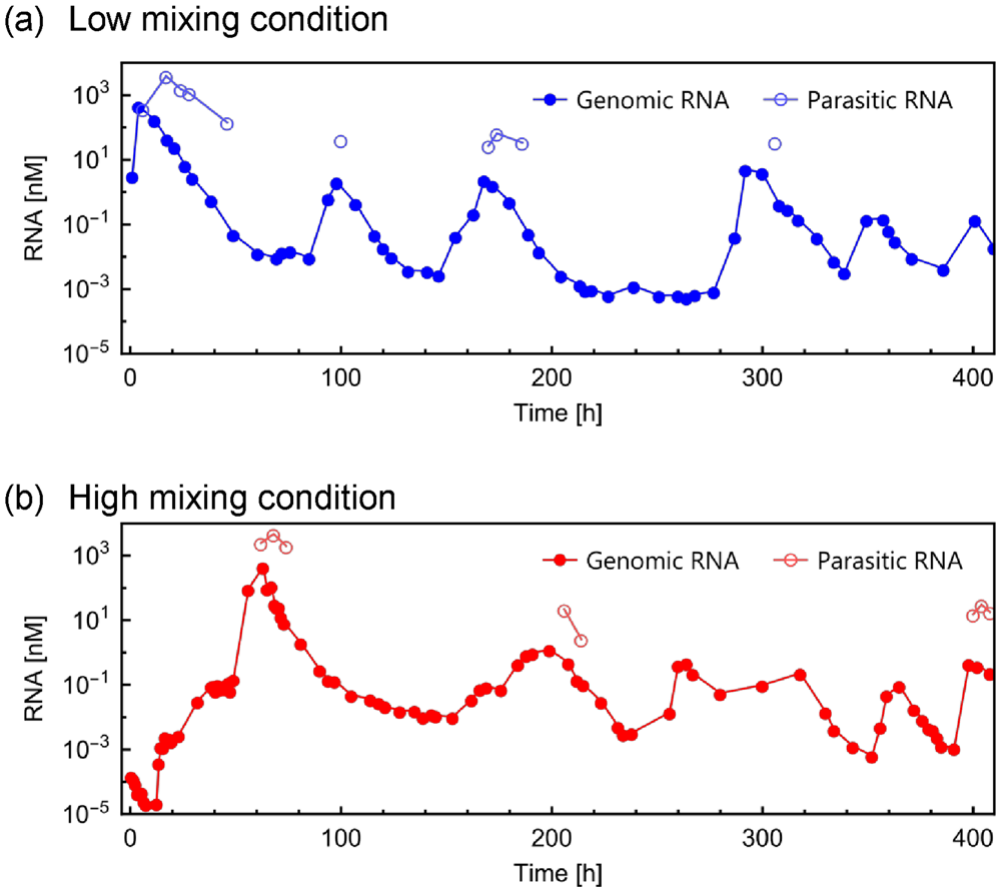
Trajectory of the average host genomic and parasitic RNA concentrations. Automated replication experiments at low. (**a**) or high (**b**) mixing intensities in the second tank. Droplets were sampled every hour for 24 hours and RNA concentrations were measured. Genomic RNA was measured by quantitative PCR after reverse transcription. Parasitic RNA concentration was measured from the band intensities after PAGE followed by staining with SYBR green II (Fig. S5). The time points without parasitic RNA concentration data indicate concentrations that are under the detection limit (approximately 3 nM).

At the low mixing rate, the trajectory of the average host genomic RNA concentration irregularly oscillated (Fig. 2a), consistent with the previous manual experiment^23^. Parasitic RNAs was also detected just after the large peak of the genomic RNA. A similar result was also observed at the high mixing rate (Fig. 2b), although the oscillation pattern was more irregular for both host and parasitic RNAs. These oscillating trajectories indicate that a similar replication process occurred in the automated droplet flow reactor as that in the manual experiments performed previously^23^.

The oscillation dynamics observed in the RNA populations can be explained by the interaction between host genomic and parasitic RNAs as described in a previous study^23^. Briefly, (1) at a low concentration of parasitic RNA, the host genomic RNA replicates and the parasitic RNA follows; (2) the parasitic RNA finally overwhelms the host genomic RNA and inhibits the replication of the genomic RNA; (3) then, both the host genomic and parasitic RNAs are decreased by dilution; and (4) after sufficient dilution of parasitic RNA, the genomic RNA can restart replication. By repeating these (1–4) processes, the oscillation dynamics in RNA populations manifest. The factors that affect the dynamics were reported in our previous theoretical study^41^.

### Sequence analysis

We analyzed the host RNA sequence at several time points during the long-term experiments. We collected the host genomic RNAs from the time point corresponding to the peaks of oscillation (46, 114, 186, 306, and 357 h for the low mixing condition, and 38, 62, 112, 206, 264, 328, 370, and 421 h for high mixing condition). After reverse transcription using specific primers, the cDNA fragments were ligated to a vector and transformed into an *E. coli* strain. The plasmids were extracted from 5–11 independent colonies at each time point and the inserted sequences were analyzed.

**Figure 3:**
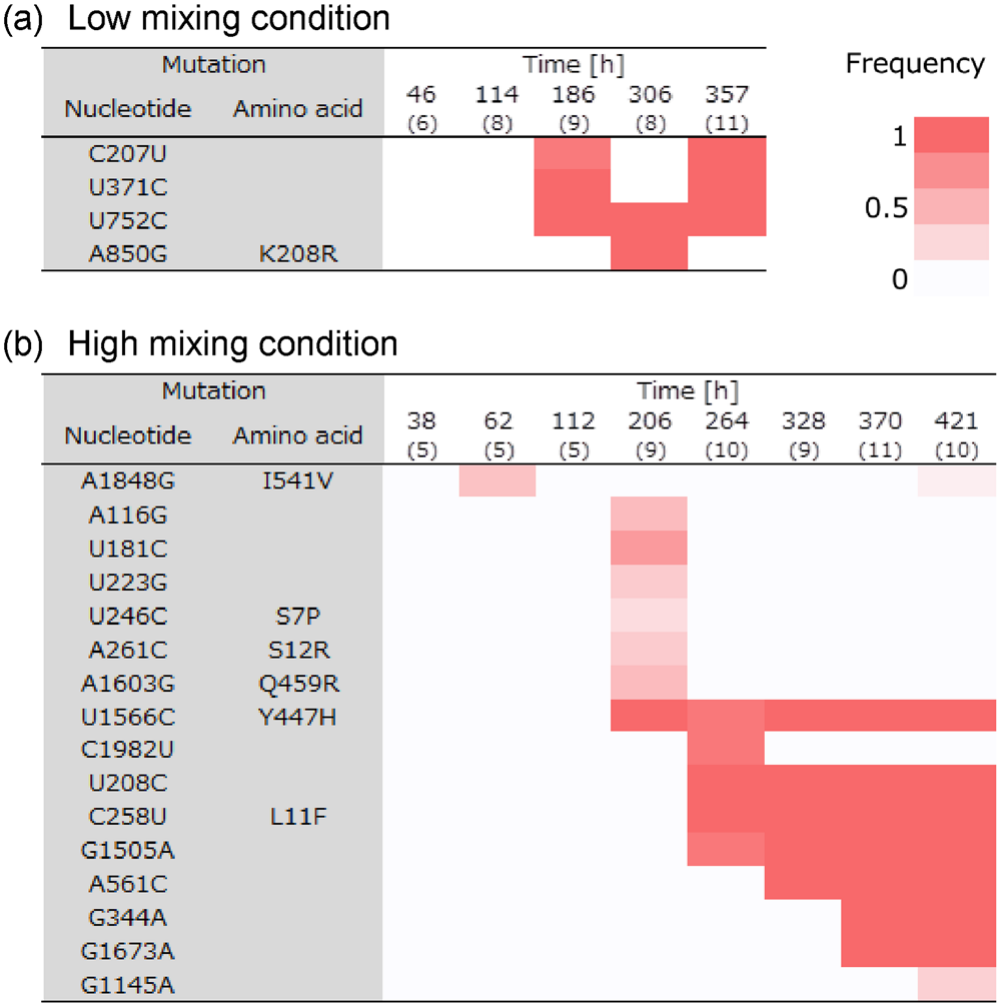
Mutations accumulated in the host genomic RNAs during the long-term replications. Mutations that were found in more than 20% of the clones sequenced at each time point were listed. The numbers of sequenced RNA clones are shown in parentheses. The frequencies are shown as a heat map for the long-term replication experiments under high (**a**) or low (**b**) mixing conditions.

Common mutations that were found in more than 20% of the clones at each time point are listed and the frequencies are represented as a heat map (Fig. 3). Under low mixing conditions, the frequencies of the mutations changed drastically during long-term replication (Fig. 3a). Three mutations (C207U, U371C, and U752C) were found in most of the genomic RNAs sequenced at 186 h, but two of them were not found at 306 h and again appeared at 357 h. At the high mixing rate, a similar temporal disappearance was also found at 62 h and 421 h for A1848G (Fig. 3b). Some mutations were found only at 206 h. Other mutations (U1566C, U208C, C258U, G1505A, A561C, G344A, G1673A, and G1145A) accumulated gradually as replication proceeded. We also analyzed the sequence data with different criteria of common mutations (i.e., more than 40%, instead of 20%), and the types and dynamics of the mutations were not significantly changed (Fig. S3).

### Assay of evolved RNAs

To analyze the properties of the genomic RNAs after evolution, we picked one mutant RNA at 357 h under low mixing conditions and 421 h under high mixing conditions as evolved RNAs, referred to as L357 and H421, respectively. These mutant RNAs contained all of the fixed mutations in the population, (i.e., C207U, U371C and U752C in L357, and U208C, U1566C, C258U, G1505A, A561C, G344A, and G1673A in H421).

**Figure 4:**
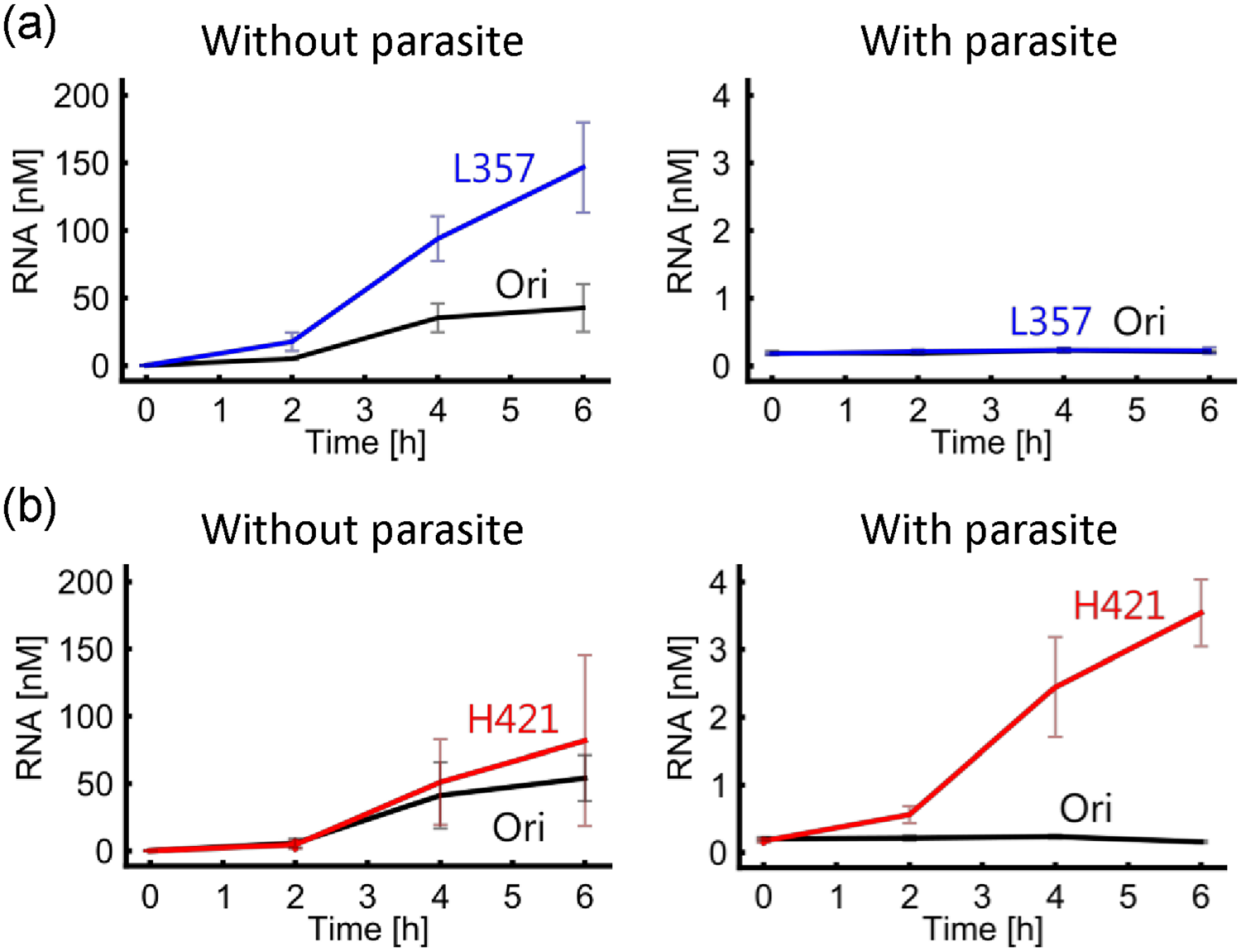
Replication assay of the evolved host genomic RNAs. The evolved genomic RNAs (L357 and H421) containing all the dominant mutations (C207U, U371C, and U752C for L357, or C208C, U1566C, C258U, G1505A, A561C, G344A, and G1673A for H421) were chosen. These evolved RNAs, L357 (**a**) or H421 (**b**), and the original host RNA (100 pM) were independently replicated with or without one of the parasitic RNAs (10 nM). The quantity of genomic RNA was measured by quantitative PCR after reverse transcription. The error bars represent standard errors (n = 3).

To compare the replication of each evolved genomic RNA (L357 or H421) with that of the original genomic RNA (Ori), we performed the translation-coupled replication experiments with each genomic RNA in the absence or presence of a parasitic RNA. L357 RNA replicated faster than the original RNA in the absence of the parasitic RNA (Fig. 4a, left), and both L357 and the original RNA barely replicated in the presence of the parasitic RNA (Fig. 4a, right). In contrast, H421 RNA replicated at a similar rate as the original RNA in the absence of the parasitic RNA (Fig. 4b, left), but much faster in the presence of the parasitic RNA (Fig. 4b, right). These results indicate that the genomic RNA selected under the low mixing conditions (L357) acquired the ability to replicate faster, while the RNA selected under high mixing conditions (H421) acquired a certain level of resistance to the parasitic RNA.

## Discussion

The automation of experimental procedures is important step to make long and laborious evolution experiments faster and easier, thus, accelerating scientific findings. However, automation has not been successfully applied to translation-coupled replication systems because these systems require cell-like compartments to link genotype and phenotype. In this study, we demonstrated automated replication and the evolution of genomic RNA in a droplet flow reactor for the first time. In previous evolutionary experiments, we manually performed a 1000 h replication in approximately 200 days. Here, we performed 400 h replications twice in a total of approximately 34 days, a much shorter duration than previous experiments. This system can be used to evaluate other self-replication systems that replicate through translation, such as the reconstituted genomic DNA replication of *E. coli*^42^ or phi29 phages^43^, or an artificial replication system composed of rolling-circle replication and recombination^5,44^. Our automated droplet flow reactor would be a useful tool to allow faster and easier *in vitro* evolution experiments involving various self-replicating systems.

In this study, we found that distinct genomic RNA mutants dominated the populations in the two evolutionary experiments. Under a low mixing condition, a mutant RNA that replicates faster dominated the population; however, under a high mixing condition, a mutant that was resistant to parasitic RNA was dominant. Since we performed only one experiment under each condition, we cannot conclude that this evolutionary result was caused by the different mixing intensities. However, the mixing intensity likely changed the evolutionary outcome since different intensities alter the propagation rates of RNAs among the droplets, which can affect the extent of the interaction between genomic and parasitic RNAs. It would be interesting to repeat these experiments to understand the relationship between the evolutionary consequences and the propagation rates of RNA.

Another interesting phenomenon that we had not previously observed was the temporal domination of mutations. Some mutations were almost fixed in the population at 186 h, undetected at 306 h, and then reappeared at 357 h. This dynamic change in mutation frequency cannot be explained by sampling error since some mutations (A850G) were also found in all of the sequenced clones (Fig. 3), indicating that the major population of the genomic RNA had changed. One possible explanation is a change in the rate of beneficial mutations due to the interactions with parasitic RNA. Parasitic RNA can also evolve in this system, and the sequences could temporally change the types of genomic RNA that are advantageous for replication. Unfortunately, we have not succeeded in cloning parasitic RNA species, likely due to the presence of strong secondary structures in the parasitic RNA; however this is not the focus of this study. Further efforts to analyze the parasitic RNA would be important to understand the evolutionary relationship between the host and parasitic RNAs.

Evolutionary processes between host and parasite must have been important for primitive life-like systems that existed prior to the origin of life, such as RNA replicators in the RNA or RNA-protein worlds. In previous literature, the possible evolutionary processes of primitive RNA replicators have been theoretically investigated and an important role of cell-like structures for the evolution of host RNA in the presence of parasite has been revealed^41,45–48^. The experimental verification of these evolutionary roles has yet to be done. The translation-coupled RNA replication system could be used as an experimental model that functionally mimics a primitive self-replicator in the RNA-protein world. In previous study, we found that the host RNA evolved parasite resistance through manual transfer experiments^23^. Here, we found that parasite resistance is not the sole consequence of evolution. To further investigate the possible evolutionary processes of a primitive host species, we need more and longer evolutionary experiments. The automated droplet flow reactor that we have established in this study provides a useful tool to experimentally investigate the long-term coevolutionary processes between host and parasitic RNAs.

The droplet reactor system we established in this study depends on the fusion and division of droplets induced by mixing. The mechanism of droplet fusion has been extensively studied to make stable emulsion^49,50^. However, the continuous fusion and division process utilized in this study is still poorly understood. For instance, the size dependency of the fusion-division rate has not been investigated. Understanding the physics of this process is important for a broader application of this equipment.

## Methods

### RNA replication system

The RNA replication system consists of an artificial host genomic RNA and the reconstituted *Escherichia coli* translation system (PURE system)^51^. As the original genomic RNA, we used the clone after the previous evolutionary experiment at Round 128, named N96(+)^17^. The host genomic RNAs were prepared by *in vitro* transcription with T7 RNA polymerase as described previously^52^. In the competition experiment (Fig. 4), S222 RNA, a 222 base RNA that spontaneously appeared during RNA replication, was prepared according to the previous study as a parasitic RNA^53^. The reconstituted translation system was prepared in our laboratory according to the procedure in the previous study^54^. The composition was listed in the Supplemental Information (Table. S1).

### Droplet flow reactor

The construction of the droplet flow reactor has been previously reported in detail^34,35^. Briefly, this system is composed of two tanks (505 μL), the inside solutions of which were mixed by magnetic stirrers (Fig. 1). The tanks were attached to electromagnetic coils to control the stirring intensity. The oil phase including mineral oil (Sigma) and surfactants (2% Span 80 (v/v, Wako) and 3% Tween 80 (v/v, Wako)) and the aqueous phase including the *E.coli* translation system were supplied into the first tank with two syringe pumps. The oil phase was used after saturation with some solutes as described previously^17^. In the first tank, droplets were prepared at 2 °C and supplied to the second tank. In the second tank, droplets contain the genomic and parasitic RNAs and fusions and divisions among the droplets were induced by mixing. Droplets in the second tank were removed at the same rate as the supply rate (dilution rate). The mixing intensities were controlled by changing the spinning period of the magnetic stirrers in the tanks. The details of the droplet flow reactor are shown in Fig. S6.

### Automated continuous replication

In this study, we performed two long-term replication experiments in the droplet flow reactor with different mixing intensities in the second tank (high or low). To change the mixing intensity, we set the mixing period (duty ratio) to 20% (5 sec mixing in 25 sec) or 5% (3 sec mixing in 60 sec) for high or low mixing conditions, respectively. The rotation frequency of the stirrers was constant (4800 rpm). The dilution rate was set at 0.2 /h (100 uL/h oil and 1 uL/h aqueous phases) during most periods for both experiments. These parameters were changed slightly at the beginning and last stage of the experiments (exact parameters are shown in Fig. S4). To initiate the reaction, we added 5 μl of the solution containing N96(+) RNA and the translation system to the second tank. The timings to refill the oil and aqueous phases are shown in Fig. S7.

The important condition for sustainable replication was low mixing intensities in the first and second tanks. As long as we kept the low (less than 5%) duty ratio in the second tank, we succeeded in performing all three independent long-term replication experiments for 400 h. The results of two of the experiments are shown in this study.

### Size distribution analysis

The RNA replication solution contained a fluorescent protein (6 µM transferrin Alexa 488, Thermo Fisher Scientific) and did not contain protein factors or genomic RNA was used as the aqueous phase. After operating the droplet flow reactor for 48 h, the size distribution of the droplets in the second tank was measured by fluorescent microscopy according to the previous study (n = 531 or 738)^34^.

### RNA measurements

The average host RNA concentrations in the droplets were measured at the indicated time by quantitative PCR after reverse transcription according to a previous study^35^. The average parasitic RNA concentrations were measured from the band intensities after polyacrylamide-gel electrophoresis (PAGE) and staining with SYBR green II using s222 RNA standard. PAGE was performed with 8% gel according to the previous study^40^. Gel images were shown in Fig. S5.

### Sequence analysis

At the time indicated in Fig. 3, genomic RNA was reverse-transcribed and PCR amplified with the following primers, Fwd: 5′-GGGTCACCTCGCGCAGC-3′ and Rev: 5′-CCGGAAGGGGGGGACGAGG-3′. The fragments were ligated with a DNA fragments PCR amplified from pUC-N96(+)^23^ (Fwd: 5′-GTCCCCCCCTTCCGGGGGGGTCCCCGGGGATCCTCTAGAG-3′ and Rev: 5′-TGCGCGAGGTGACCC-3′) and transformed into *E. coli* JM109 strain. Plasmids were extracted from 5–11 colonies at each time and sequenced.

### Competition experiment

The original N96(+) RNA, and each of the evolved RNAs (L357 or H421) (100 pM) was mixed with the reconstituted translation system with or without 10 nM S222 RNA (parasitic RNA) and incubated at 37 °C for 6 h. At the times indicated in Fig. 4, the RNA concentrations were measured as described above.

### Data availability

All data generated or analysed during this study are included in this published article (and its Supplementary Information files).

## Electronic supplementary material

Supplementary Information
