# Supplementary material for: Automated in vitro evolution of a translation-coupled RNA replication system in a droplet flow reactor
Source: Sci Rep. 2018 Aug 8;8:11867. doi: 10.1038/s41598-018-30374-0 (PMC6082869; doi:10.1038/s41598-018-30374-0)
Supplement: Supplementary file 1 — Supplementary Information [file 41598_2018_30374_MOESM1_ESM.pdf]

Supplementary Information

Automated *in vitro* evolution of a translation-coupled RNA replication system in a droplet flow reactor

Tomoaki Yoshiyama, Tetsuo Ichii, Tetsuya Yomo, Norikazu Ichihashi

Table S1. Composition of the translation system

Names of factors	Concentration	Names of factors	Concentrati on
Initiation factor 1	25 μM	tryptophanyl-tRNA synthetase	28 nM
Initiation factor 2	1 μM	tyrosyl-tRNA synthetase	150 nM
Initiation factor 3	4.9 μM	valyl-tRNA synthetase	17 nM
Elongation factor G	1.1 μM	methionyl-tRNA formyltransferase	590 nM
Elongation factor Tu	80 μM	myokinase	1.4 μM
Elongation factor Ts	3.3 μM	creatine kinase	250 nM
Release factor 1	49 nM	nucleoside diphosphate kinase	16 nM
Release factor 2	48 nM	pyrophosphatase	41 nM
Release factor 3	170 nM	Trigger factor	1 μM
ribosome recycling factor	3.9 μM	*E. coli* DEAH type RNA helicase A *	100 nM
alanyl-tRNA synthetase	730 nM	ribosomes	1 μM
arginyl-tRNA synthetase	31 nM		
asparaginyl-tRNA synthetase	420 nM	tyrosine	0.3 mM
asparagyl-tRNA synthetase	120 nM	cysteine	0.3 mM
cysteinyl-tRNA synthetase	24 nM	18 other amino acids	0.36 mM
glutaminyl-tRNA synthetase	60 nM	tRNA mix (Roche),	0.39 mg/ml
glutamyl-tRNA synthetase	230 nM	ATP	3.75 mM
glycyl-tRNA synthetase	86 nM	GTP	2.5 mM
histidyl-tRNA synthetase	85 nM	CTP	1.25 mM
isoleucyl-tRNA synthetase	370 nM	UTP	1.25 mM
leucyl-tRNA synthetase	41 nM	N-2-hydroxyethylpiperazine-N'-2-ethanesulfonic acid (pH 7.6)	100 mM
lysyl-tRNA synthetase	120 nM	glutamic acid potassium salt	70 mM
methionyl-tRNA synthetase	110 nM	spermidine	0.375 mM
phenylalanyl-tRNA synthetase	130 nM	magnesium acetate	16 mM
prolyl-tRNA synthetase	170 nM	creatine phosphate	25 mM
seryl-tRNA synthetase	78 nM	dithiothreitol	6 mM
threonyl-tRNA synthetase	84 nM	5-formyl-5,6,7,8-tetrahydrofolic acid	10 μg/ml

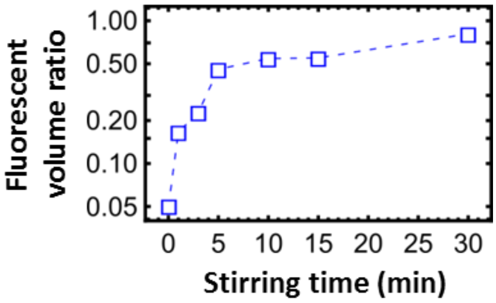

Figure S1. Propagation of fluorescent dye among droplets

After running the automated droplet reactor for 48 h under the same condition as the low mixing condition, 1/20 of fluorescent droplets containing 6 μM Transferrin Alexa Fluor 647 (Invitrogen) was added in the second tank and the propagation of fluorescence among the droplets was measured according to the previous study[34], at the same mixing intensity (5% duty ratio) without supplying new droplets.

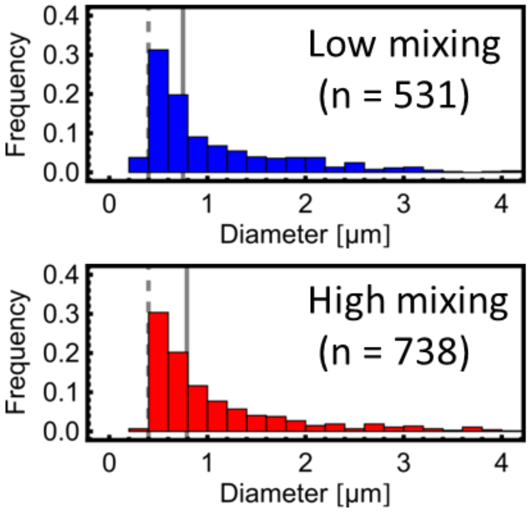

Figure S2 Size distributions of the droplets

Droplet sizes were measured by microscopy as described in the Materials and Methods section. The average sizes are indicated with the solid lines.

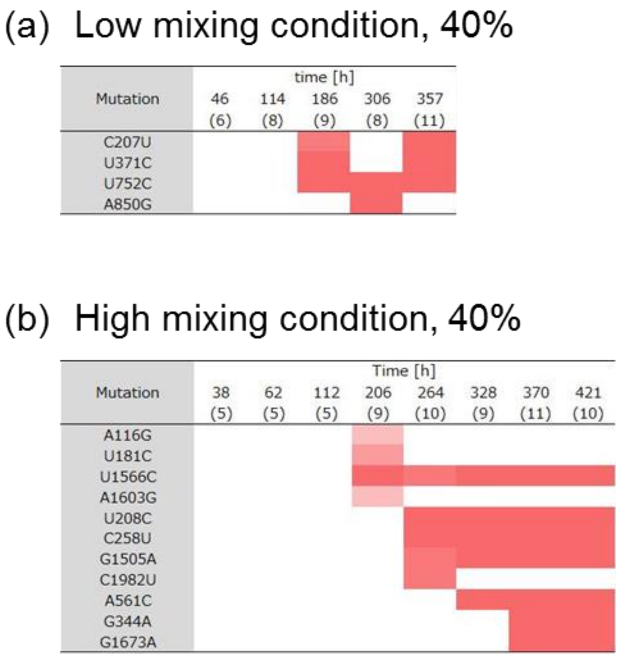

Figure S3. Mutations accumulated in the host genomic RNAs during the long-term replications obtained with different criteria.

Mutations that were found in more than 40% of the clones sequenced at each time point were listed. The numbers of sequenced RNA clones are shown in parentheses. The frequencies are shown as a heat map for the long-term replication experiments under low (a) or high (b) mixing conditions.

Low mixing condition

Reaction time (h)	Dilution rate (/h)	Duty ratio in the second tank (%)
0 - 421	0.2	5

High mixing condition

Reaction time (h)	Dilution rate (/h)	Duty ratio in the second tank (%)
0 – 6	0.5	100
6 – 8	0.5	20
8 – 12	0	0
12 – 41	0.1	20
41 – 378	0.2	20
378 - 421	0.25	20

Figure S4. Parameters of the two long-term experiments

For the high mixing condition, we adjusted the dilution rate and the duty ratio in the second tank to stabilize RNA replication in the early stage (prior to 41 h).

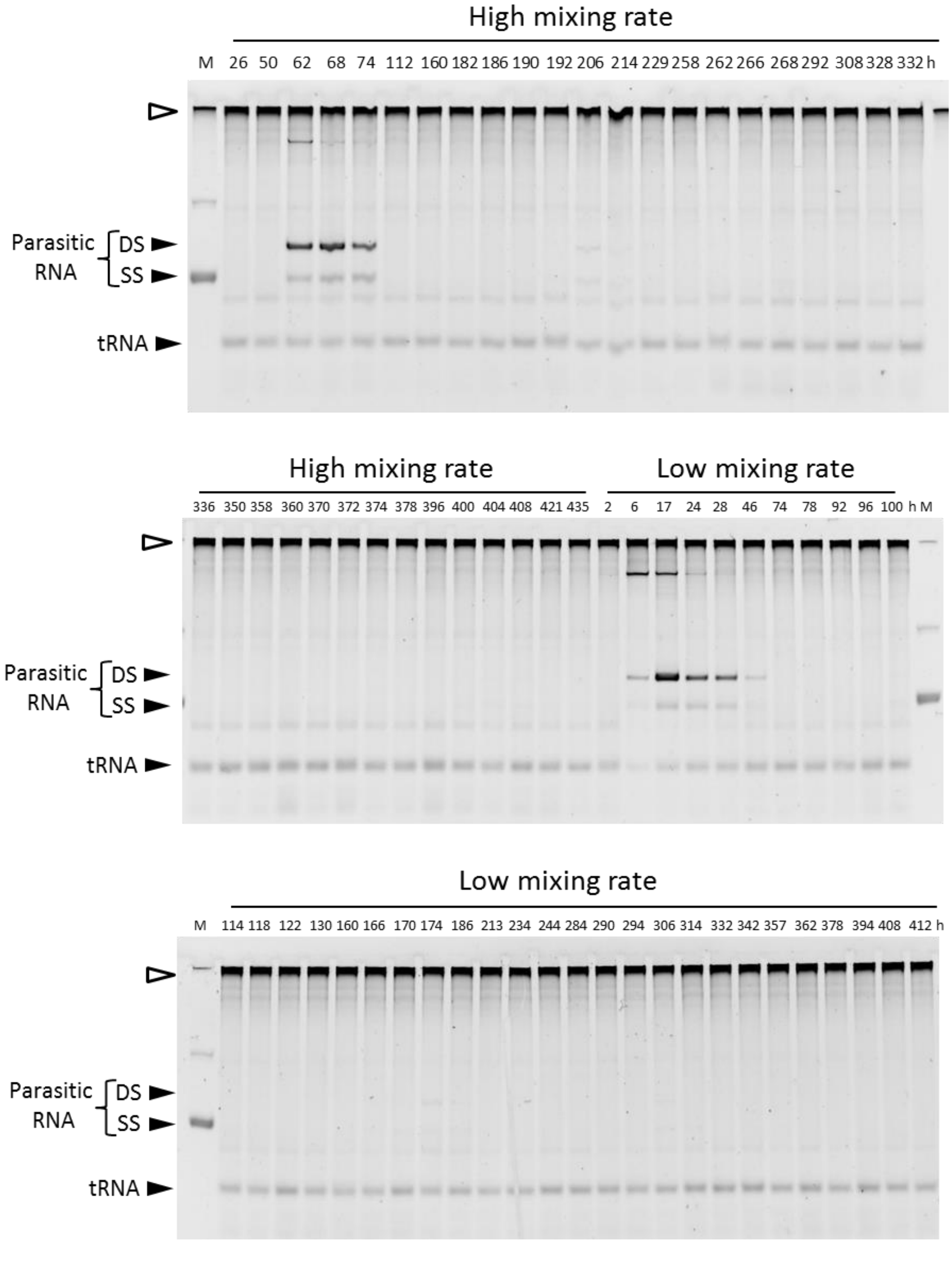

Figure S5. Polyacrylamide-gel analysis of parasitic RNA.

The total RNAs of the reaction mixtures, including the host genomic and parasitic RNAs, tRNAs, and rRNAs, were purified from the droplets at the indicated time points using an RNAeasy column (QIAGEN). The total RNA (250 ng) was applied in a lane along with a standard parasitic RNA (S222 RNA, 250 fmol, indicated as "M"). The location of the single or double strands of the parasitic RNA (SS or DS) is indicated. The host RNAs were not sufficiently separated from rRNA under these conditions and appeared at the top of the gels (indicated with white arrowheads).

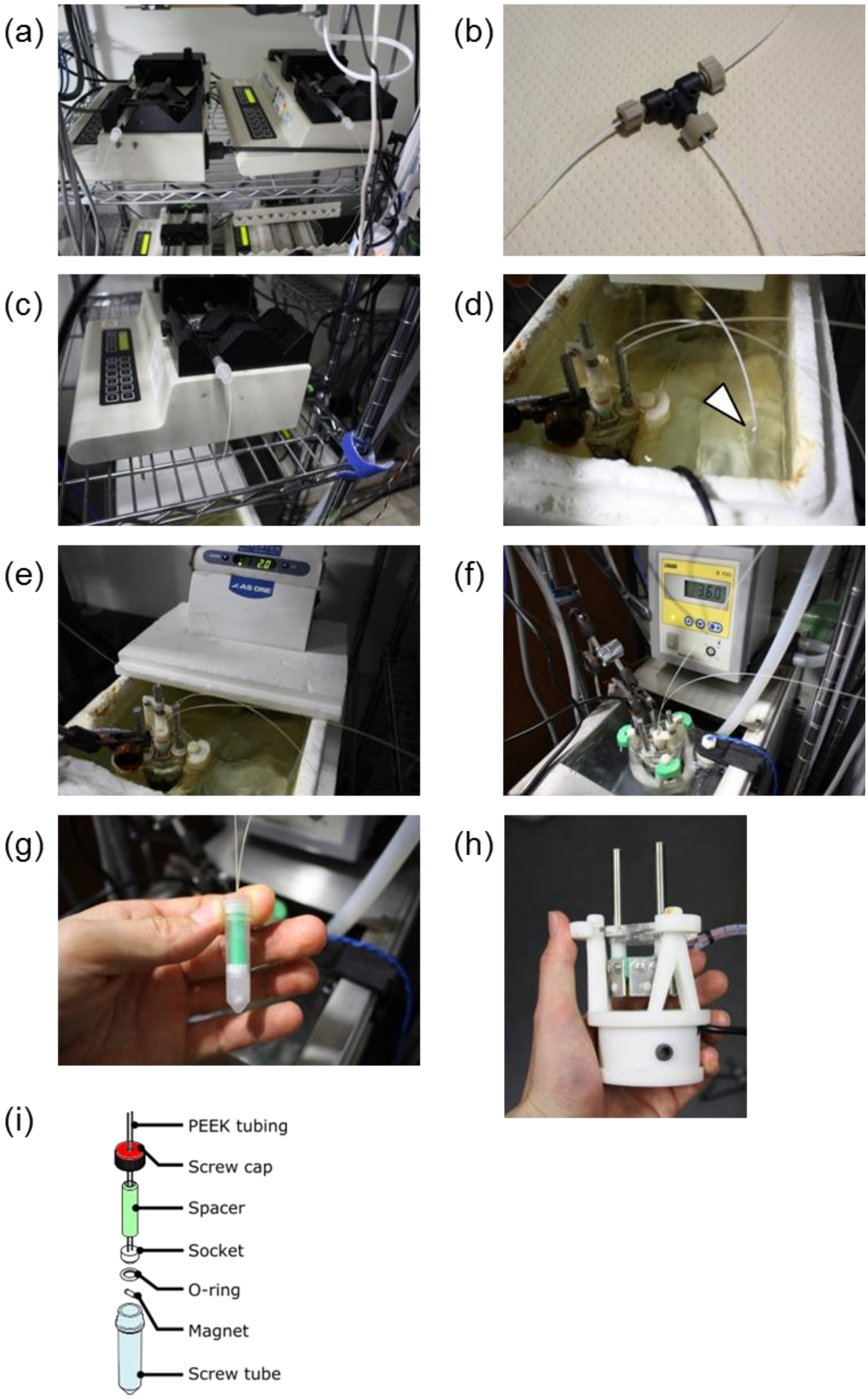

Figure S6. Details of the droplet reactor.

(a) Syringes and pumps for supplying the oil phage: We used two sets of pumps (SP210iw, World Precision Instruments) and syringes (1002TLL, Hamilton) to supply

the oil phases. The two syringes were connected with a connector (P-727, Upchurch Scientific) shown in (b). The syringes containing the oil phase were stored at 25 °C. We added new oil phages every 1-2 days. (c) The syringe and pump for supplying the aqueous phase (the translation system): The syringe (1705TLL, Hamilton) contained 50 μL of another oil (FC-40, 3M) and was connected to a tube containing 50 μL of the aqueous phage. We changed the aqueous phase every 1-2 days. (d) To avoid the inactivation of the translation system, the tube containing the aqueous phase was submerged in the water bath at 2 °C (indicated with the arrowhead). PEEK tubing (outer diameter 0.51 mm and inner diameter 0.125 mm) and PTFE tubing (outer diameter 1.5 mm and inner diameter 0.5 mm) were used. (e) 1st tank, sunk in a water bath at 2 °C to avoid the inactivation of the translation system. (f) 2nd tank, sunk in a water bath at 36 °C to promote the translation and RNA replication. Each tank consisted of the inside solution container (g) and outside magnetic stirrer (h). (i) The constitution of the inside solution container: The container consists of 2-mL conical tube with screw cap (1392-200, Watson), a magnet stirrer (1-6618-01, AS ONE), a silicone O-ring (SI50 S-6, SAKURA SEAL), a PTFE socket, a spacer. The container of the 1st tank has three tubes (those that supply the oil or aqueous phase, and one that connects to the 2nd tank). The container of the 2nd tank has two tubes (one to connect to the 1st tank and one to the outlet).

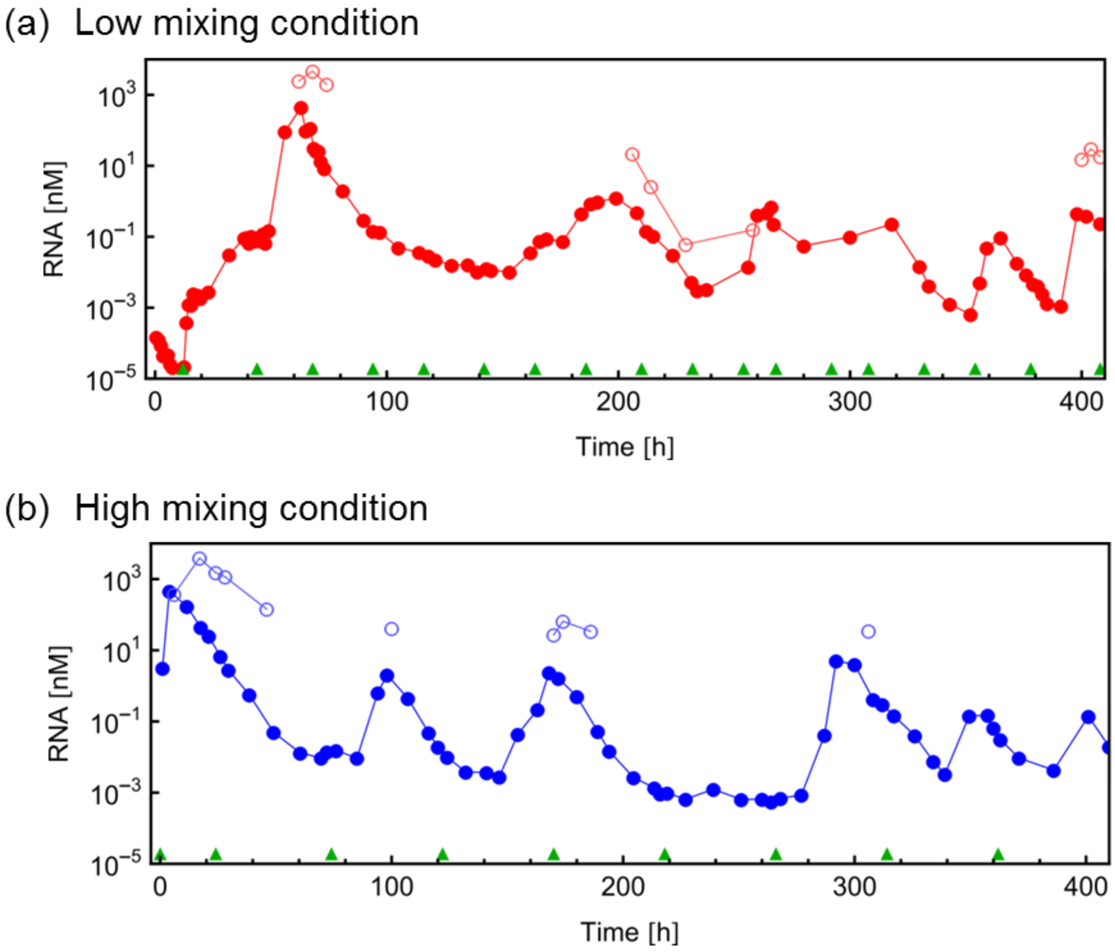

Figure S7. Refill timings of the oil and aqueous phases.

The oil and aqueous phases were refilled at the times indicated with green triangles.
